# Normative framework of informed consent in clinical research in Germany, Poland, and Russia

**DOI:** 10.1186/s12910-021-00622-6

**Published:** 2021-05-01

**Authors:** Marcin Orzechowski, Katarzyna Woniak, Cristian Timmermann, Florian Steger

**Affiliations:** Institute of the History, Philosophy and Ethics of Medicine, Ulm University, Parkstraße 11, 89073 Ulm, Germany

**Keywords:** Biomedical research, Informed consent, Ethics, Medical legislation, Germany, Poland, Russia

## Abstract

**Background:**

Biomedical research nowadays is increasingly carried out in multinational and multicenter settings. Due to disparate national regulations on various ethical aspects, such as informed consent, there is the risk of ethical compromises when involving human subjects in research. Although the Declaration of Helsinki is the point of reference for ethical conduct of research on humans, national normative requirements may diverge from its provisions. The aim of this research is to examine requirements on informed consent in biomedical research in Germany, Poland, and Russia to determine how each national regulatory framework relates to the provisions of the Declaration of Helsinki.

**Methods:**

For this analysis, we conducted a search of the legal databases “Gesetze im Internet” for Germany, “Internetowy System Aktow Prawnych” for Poland, and “ГAPAHT – Garant” for Russia. The search was complemented by a review of secondary literature contained in the databases Google Scholar, PubMed, Polish National Library, and eLibrary.ru. We have identified 21 normative regulations containing provisions on informed consent in clinical research in all three countries. The content of these documents was systematically categorized and analyzed.

**Results:**

The normative framework in all three countries shows a strong commitment towards the core ethical principles of research envisaged in the Declaration of Helsinki. Nevertheless, provisions on informed consent vary between these three countries. The differences range from the method and language in which information should be provided, through the amount of information required to be disclosed, to the form of documenting consent or withdrawal. In the case of research on vulnerable groups, these differences are particularly visible.

**Conclusions:**

The identified differences can negatively impact the ethical conduct of international clinical studies. Attention needs to be paid that flexibilities within national regulations are not misused to undermine the protection of research subjects. Achieving global or regional legislative harmonization might prove impossible. Such lack of legal consensus reinforces the significance of the international ethical agreements.

*Trial registration*: Not applicable.

## Background

Informed consent (IC) in biomedical studies can be defined as a process of communication involving both investigator and research participant that culminates in the authorization or refusal of participation in a research study [1]. It is grounded in basic principles of human dignity, patient autonomy, and individual assessment of risks and benefits. IC in research is built on three constructs: study information, participant’s comprehension and understanding, and voluntary participation [2]. Its status as a central pillar of biomedical research and practice is recognized in several international documents, such as the Nuremberg Code (1947), the Declaration of Helsinki (1964) with its subsequent updates, and the Belmont Report (1979).

Today, biomedical research, especially pharmaceutical research, is increasingly carried out by companies in offshoring mode. Countries in Central and Eastern Europe may constitute an inviting research environment due to centralized hospital systems and relatively fast patient recruitment. The ClinicalTrials.gov database of privately and publicly funded clinical studies lists 1.257 clinical trials that started in 2019 in Germany. These numbers for Poland and Russia are 552 and 372 respectively [3]. The EU Clinical Trials Register counts the same year a lower number for Germany (938) and a somewhat higher number of applications for Poland (560) [4]. Many of these trials are being conducted as multicenter studies, often in several countries simultaneously. For example, there were 143 multicenter studies conducted in 2019 between Germany and Poland [4]. People of different nationalities participate in these studies. Any potential restrictions depend on research design and study protocol. For foreigners and citizens, applicable are regulations of the country in which the particular trial takes place.

There is a risk that ethical compromises will occur in such contract studies [5]. This is mostly due to inadequate knowledge of the respective legislation and the socio-cultural aspects of medical ethics. Research teams need to be aware of the prevailing cultures in the study country and their relation to national regulations. Cultural values influence the realization of human subjects’ rights, the communication of information, or the protocols for randomization and the use of placebo [6]. Different lines of historical development as well as various social and cultural values and orientations affect the interpretation of international ethical guidelines [7]. Furthermore, after assessing national circumstances, policy-makers often opt to establish additional safeguards and allow certain flexibilities. As a result, divergent normative regulations can arise in individual countries, even at the subnational level in federal countries, and include further variations when implemented in medical practice. This is particularly evident in the area of informed consent in medical research on human subjects [8].

The Declaration of Helsinki [9] is generally perceived as the international reference point providing common guidelines for ethical conduct of research on humans. Despite its legally non-binding character, there is a strong commitment—particularly among medical associations and ethics committees—to adhere to the principles of the Declaration of Helsinki. The ethical guidelines of the Declaration have an impact on research practice that goes beyond the legal sphere. Even so, there can be a significant gap between the principles of the Declaration and national provisions. The Declaration foresees such possible discrepancies, for instance when it states that physicians should consider the ethical, legal, and regulatory norms for research on humans in their own countries (§10). Yet at the same time, the Declaration specifies in the mentioned paragraph that no national norms should reduce or eliminate any of its protections. Therefore, policy-makers have a certain leeway to specify or elaborate on the provisions of the Declaration in consideration of national circumstances, as long as these do not depart or contradict the main principles.

The aim of this research is to examine the requirements on informed consent in biomedical research in Germany, Poland, and Russia to determine how each national regulatory framework relates to the provisions of the Declaration of Helsinki. These three countries have been chosen as the focus of the investigation for several reasons. First, they are among the countries conducting a high number of clinical research each year. Second, they belong to supranational organizational structures, i.e. European Union (EU) and Eurasian Economic Union (EAEU), which require adaptation of national laws to common regulations. Third, we hypothesize that despite different historical developments these countries share sufficient socio-cultural values and orientations, to have enacted similar regulations on IC.

## Methods

This investigation is a comparative analysis of the regulations on informed consent in research on human beings in Germany, Poland, and Russia. The relevant regulations were searched in the national databases: “Gesetze im Internet” for Germany, “Internetowy System Aktów Prawnych” for Poland, and “ГAPAHT – Garant” for Russia. The search in German and Russian databases was conducted with the use of pre-selected keywords: “informed consent” and “clinical research” (German: “informierte Einwilligung”, “klinische Prüfung”; Russian: “инфopмиpoвaннoe coглacиe”, “клиничecкoe иccлeдoвaниe”). The search in the Polish database was conducted with the use of keywords provided by the database “research and certificates”, “health protection”, “pharmaceutical law”, “medical devices”, “pharmaceutical products” (Polish: “badania i certyfikacje”, “ochrona zdrowia”, “farmaceutyczne prawo”, “medyczne wyroby”, “farmaceutyczne środki”). In addition, examined were relevant scientific texts in English, Polish, and Russian, retrieved from the databases: Google Scholar, PubMed, the Polish National Library (PBN), and eLibrary.ru to provide contextual background and identify relevant documents not detected through our search in the legal databases.

The investigation was designed as a documentary research with legal documents and professional guidelines as source materials. As official texts, they meet all of the four criteria for documentary analysis: authenticity, credibility, representativeness, and meaning [10]. Identified legal documents with relevant titles were examined in detail to identify the provisions pertaining to the research topic. The content of the documents was manually analyzed, systematically categorized, and described according to the predefined topics.

We identified 21 documents with information pertaining to the research aim. These include binding legal acts, such as laws, regulations, or resolutions as well as legally non-binding guidelines on medical professional ethics. The documents were analyzed in their original language and in versions containing all amendments that occurred before August 1, 2020. An outline of the documents included in the analysis is provided in Table 1.

**Table 1:** Analyzed national regulations and professional guidelines

Country	Name of the legal document	Sections	Date of introduction
Germany	Basic Law for the Federal Republic of Germany [11]	Art. 1	May 23, 1949
	(Model) Professional Code for Physicians in Germany [12]	Art. 15	December 14, 1998
	Medical Devices Act [13]	Art. 20-22	August 7, 2002
	Regulation on the Application of Good Clinical Practice in Conducting Clinical Trials with Medicinal Products for Use in Humans [14]	Art. 3	August 9, 2004
	Medicinal Products Act [15]	Art. 40-42	December 12, 2005
	Regulation on Clinical Investigations with Medical Devices [16]	Art. 3	May 10, 2010
	Radiation Protection Act [17]	Art. 36	June 27, 2017
	Radiation Protection Regulation [18]	Art. 133-138	November 29, 2018
Poland	Medical Code of Ethics [19]	Art. 43-44	May 3, 1992
	Act on Professions of Doctor and Dentist [20]	Art. 25	December 5, 1996
	Constitution of the Republic of Poland [21]	Art. 39	April 2, 1997
	Pharmaceutical law [22]	Art. 37a-37ia	September 6, 2001
	Medical Devices Act [23]	Art. 40-41	Mai 20, 2010
	Regulation on Good Clinical Practice [24]	Art. 7	Mai 2, 2012
Russia	Constitution of the Russian Federation [25]	Art. 21.2	December 12, 1993
	Ethical Code of Russian Physician [26]	Art. 18	November 1, 1994
	Law on Circulation of Drugs [27]	Art. 43	April 12, 2010
	Law on Basics of Health Protection of the Citizens in the Russian Federation [28]	Art. 36.1	November 21, 2011
	GOST R ISO 14155-2014 Clinical Investigations. Good Clinical Practice [29]	Art. 4.7	June 1, 2015
	Regulation on the Procedure for Giving Informed Voluntary Consent to Medical Care in the Framework of Clinical Testing [30]	Art. 2-5	July 21, 2015
	Regulation on the Approval of the Rules of Good Clinical Practice [31]	Art. 52-56	April 1, 2016

## Results

An analysis of the regulations concerning IC shows that in each country the subject is specified in several different documents, which may increase uncertainty regarding the proper conduct of clinical studies.

In Germany, Poland, and Russia, the principle of IC for conducting studies on human subjects is written down in the fundamental state laws. The Polish Constitution provides in Art. 39 for voluntary consent for every medical experiment: “No one shall be subjected to scientific experimentation, including medical experimentation, without his voluntary consent” [21]. Similarly, the Russian Constitution provides in Art. 21, point 2 for mandatory declaration of consent in any research with human subjects: “Nobody may be subjected to medical, scientific or other experiments without voluntary consent” [25]. The Basic Law for the Federal Republic of Germany does not contain an explicit paragraph on informed consent; however, this principle can be derived from the principle of absolute protection of human dignity laid down in Art. 1 of the Basic Law [11].

The normative framework in all three countries shows a strong commitment towards the core ethical principles of research envisaged in the Declaration of Helsinki. Differences in the regulations on IC can be found in the areas of method and content of participant information, the form of consent and its withdrawal, consent in emergency situations, and obtaining approval for participants incapable of giving consent and minors (Table 2).

**Table 2:** Differences in regulations on informed consent in Germany, Poland and Russia

	Germany	Poland	Russia
**Content of information**			
Scope of information	Selected information	Full information	Full information
**Method of information**			
Language used	German binding, additional languages for comprehension facilitation	Polish	Russian
**Form of consent and its withdrawal**			
Verbal consent requirements	One witness	Two witnesses	One witness
Withdrawal of consent	Written or verbally	Not specified	Not specified
**IC in research with vulnerable groups**			
Participation of incapacitated persons	Assent required if feasible Consent required from legal representative	Assent required if feasible Consent required from legal representative A guardianship court can override the decision of the legal representative	Consent required only from legal representative
Participation of minors	Assent required if feasible Consent required from legal representative Withdrawal by expression of will	Assent required if feasible Consent required from legal representative Withdrawal if able to understand the situation	Inclusion not foreseen Consent required only from legal representative
Participation in emergency situations	Subsequent consent required as soon as possible	Not specified	Not specified

### Content of information

Concerning the content of participant information, all three countries regulate the provision of information on aims, methods, conditions, as well as the scope and risks of the trial. Participants of a clinical trial need to also receive information on the purpose and scope of processing personal data. Moreover, participants should receive information that they have the right to withdraw from the study at any time. Such formulations follow the provisions of the Declaration of Helsinki. German legal regulations require a relatively short list of information items that should be provided to the participant. These should comprise the essence, meaning, and scope of the clinical trial [13–15, 18], purpose and scope of processing personal data [14, 15], and, if applicable, the use of radioactive material [18]. In the case of Poland and Russia, the catalog of topics is considerably longer. It includes, among others, information on the estimated number of participants in the study, duties of participants, financial claims in case of damages inflicted during the trial, and the type of compensation for participation. In addition, participants should receive confirmation that they will be notified on all new information that may affect their decision to continue to participate [24, 31].

### Method of information

The Declaration of Helsinki states that potential subjects must be adequately informed (§26). There are no specifications on whether the communication with the patient should occur in writing or verbally. Regulations in all three countries provide little specification on the method of participant information. In each country, the person responsible for providing information is the lead researcher or a member of the research team [13–15, 18, 23, 29]. Only German legal regulations specify that in clinical studies the information needs to be communicated by a physician [15]. In Poland, a special consultant can be appointed to provide additional information about the clinical study and to inform about the rights of the subject [24]. German legal regulations require verbal communication with the participant; the provision of written information is not explicitly mentioned. However, consent should be given after receiving appropriate documentation, which indicates that some written information should also be made available [14, 18]. In the specific case of research with radioactive materials, information must be provided in writing [18]. Polish and Russian legal regulations require both verbal and written information [22, 24, 27, 31]. Only German norms explicitly state that information can be additionally given in the participant’s native language [16], Polish and Russian regulations do not mention the language in which provision of information should occur. To improve understanding, Russian regulations stipulate that information should be provided in simple and non-technical language [29, 31].

### Form of consent

In all three countries IC should preferably be documented in written form through the dated participant’s signature. This preference corresponds to the provisions of the Declaration of Helsinki. In special situations, when providing written consent is impossible, e.g. due to the inability to write, verbal consent is acceptable (§26). Verbal consent should be given in presence of one witness in Germany and Russia [14, 15, 29] or two witnesses in Poland, and recorded as such in the protocols [20, 22]. Withdrawal of consent for participation in the trial may occur at any time. It can be given verbally or in writing in Germany [13, 15, 18]. Polish and Russian legal regulations do not specify the form in which consent can be withdrawn. Only in Germany it is possible to provide consent, and to withdraw it, in an electronic form [13, 15, 18].

### IC in research with vulnerable groups

Special precautions need to be taken to protect vulnerable groups during research. To this group belong participants who are physically or mentally incapable of giving consent, and children and adolescents before the legal age of consent. The Declaration of Helsinki includes ethical norms prescribing the process of IC with these particular subjects (§28–30). Regulations in all three countries explicitly address the issue. Participation in research of persons incapable of giving informed consent requires approval from their legal representative [13, 15, 20, 22, 29]. However, in Germany, assent needs also to be given by the represented person, provided that this person is capable of understanding the nature, significance, and implications of the clinical investigation and is able to form a rational intention in light of these facts [13]. In Poland, a legally incapacitated person is required to provide written assent if he or she is able to consciously express their opinion [22]. When this is not the case, consent can be given by the legal representative. If the legal representative refuses to give consent, a guardianship court can overrule this decision if it is deemed in the best interest of the patient [19, 20]. Guardianship courts may also give consent in case of participants with the capacity to act in law but unable to consciously express their opinion [20, 22]. Withdrawal of consent in Germany may occur through the stated refusal of the legal representative, or through any expression of the participant’s will. In Poland, in addition to the legal representative, the incapacitated participant may withdraw from the study if he or she is able to express an opinion and evaluate information about the study [20, 22, 23]. In contrast, Russian regulations do not provide for the inclusion of incapacitated participants in the process of obtaining informed consent. Information should be presented to their legal representatives or guardians appointed by a federal agency. These legal representatives provide written consent or decide about withdrawal from the clinical study [27–29].

The second vulnerable group expressly mentioned in context of IC in research in the normative framework of all three countries are minors. In Germany and Poland, the study information should be provided to legal representatives by a member of the research team. In Germany, in clinical studies with minors, the information needs to be communicated by a physician [15]. The minor should be informed, by a person who is experienced in communicating with minors [13, 15, 22, 23]. The content of the information should be provided in a language understandable by the minor and adjusted to the minor’s apprehension capacity [13, 22, 23]. In Germany, consent should be given by both legal representatives or authorized representatives in view of the minor’s presumed will, where such will can be ascertained [13, 15]. If the minor is able to comprehend the nature, significance, and implications of the clinical trial, his or her assent is also required. In Poland, written assent is to be given by the legal representative and by minors, who are older than 16 years [20, 22, 23]. Minors younger than that age can also give their assent, provided that they are able to express their own opinion. In both Germany and Poland, withdrawal of consent occurs through a declaration expressed by the legal representative. Furthermore, in Germany, the minor may refuse participation through an explicit declaration or any other form of expression of the will [15]. In Poland, the minor can withdraw the consent if he or she is able to express an opinion and evaluate information on the clinical trial [20, 22, 23]. In contrast, Russian regulations do not foresee the inclusion of minors in the informed consent process. Information on the clinical study should be provided to the parent, legal representative, or a guardian appointed by a federal agency, who provides written consent for the study and has the right to withdraw the participation of the minor [27, 28, 30].

In emergency situations, participation can start immediately in research that can save life, restore good health, or alleviate the suffering of the person concerned. According to the Declaration of Helsinki, consent must be obtained as soon as possible (§30). In such a case, German law requires obtaining a subsequent IC as soon as it is possible and reasonable [15]. Obtaining retrospective consent for treatment in an emergency situation is not specified in Polish and Russian legal regulations.

## Discussion

Although the Declaration of Helsinki is not legally binding in the context of international law, its impact on research practice, ethical reasoning and national legal regulations remains undisputed in Germany, Poland, and Russia. Through the inclusion in medical Codes of Ethics in all three countries, the Declaration gains binding character. The content of the Declaration constitutes therefore, beside ethical guidance, applicable regulation for medical researchers. It is also noticeable that many normative regulations regarding IC in all three countries adopt the ethical foundations provided by the Declaration without expressly mentioning it. However, the results of our comparative analysis show that provisions on IC vary among the countries, especially in the areas that are not explicitly covered by the Declaration. The differences range from the method of communication with participants, through the amount of information that they are required to receive, the form of providing consent or withdrawal, to the procedure of IC in research with vulnerable subjects.

### Content of information

The Declaration of Helsinki lists the catalogue of information that should be provided to participants. However, it also expressly recognizes that information should be tailored to the individual needs of potential subjects. Clearly stated research objectives and methods presented by trained recruiters can positively influence participation in the study and decrease the level of uncertainty about the study’s aims and methods [32]. None of the analyzed regulations explicitly ask to provide specific, person-oriented information, tailored to the participant’s needs. Professional research ethics recommends that researchers should strive to find out what types of information are likely to be relevant to those who consider participating in research [33]. Regarding the long catalog of issues that need to be disclosed according to the Polish and Russian regulations, the typical information process may be overwhelming for participants [34]. Here, German regulations reveal a preference for keeping the information participants receive short to increase the chances that the key points of the procedure are adequately understood. Narrowing down the information given to participants can however reflect the priorities of those selecting the information, which may not necessarily be shared with those of the participant. Regulations in Poland and Russia opt to provide the patient with more information, preferring to risk overwhelming some participants in order to make sure patients have indeed all information they may value. It has also been pointed out that the provision of extensive information in Poland has the aim of protecting researchers from possible damage claims [35].

### Method of information

Inadequate understanding of the information provided in the process of IC is a common phenomenon observed in research and other areas of medicine [36]. Therefore, central is the issue of an effective and adequate information process. Employment of one-time information meetings or extensive information sheets is unlikely to contribute to the proper process of consent [34]. Especially in the case of research that involves complicated interventions or complex technologies, participants should be able to understand the procedures and their implications. Despite the need to adapt to new research circumstances, the regulations in all three countries do not mention the possibility to amend IC protocols by using for example dynamic consent, which requires continuous communication of new developments and subsequent approval [37]. Using such a type of consent is increasingly important for several fields of medical research, e.g. next-generation genetic sequencing or research with the use of a donor’s genetic material in studies on induced pluripotent stem cells (iPSCs) [38]. An additional method to inform patients worth exploring is the use of multimedia, social media, and other interactive tools. Despite some disadvantages connected to the use of social media in research settings, they constitute a valuable tool of communication and interaction between researchers and participants [39]. However, this method should only be complementary to information communicated in person by a researcher or a physician.

Ensuring that the subject has understood the information is central for an autonomous decision and explicitly stated as a principle in the Declaration of Helsinki (§26). Despite this, regulations in all three countries do not provide a requirement for evaluating the level of comprehension of the provided information. Comprehension can be assessed through a questionnaire, interviews or, if study resources allow it, by a third party not affiliated with the project [40].

To improve understanding, it is nowadays important to also pay attention to language barriers. Only German regulations explicitly state that translated information can be provided in the native language of potential subjects. This mirrors the progressing heterogeneity of the German society. However, for legal purposes, patients still need to sign the binding German text version. Considering the similar ethnic and cultural diversity within Russia and the changing societal composition in Poland, such omission is a risk for patient autonomy and can be a discriminating factor. It may lead to situations in which a proper processing of information is not guaranteed. In this respect, clear guidelines need to be adopted to face the communication challenges of a modern society.

### Form of consent and its withdrawal

Regulations in all three countries follow the provisions of the Declaration of Helsinki regarding written or verbal form of consent. However, only Germany makes it possible to legally record in electronic form the provision or withdrawal of consent to participate in a study. Such flexibility gains importance with geographical distance between the research center and the research subject or under time pressure. Also during the ongoing pandemic, for instance, the possibility of recording the consent through means other than a handwritten signature becomes crucial to reduce the risk of contagion [41]. Alternative forms to register consent should be made available to protect research staff, participants, and those processing the documents. For example, as a response to the pandemic, the US Food and Drug Administration has formally endorsed telephonic and electronic consent as an alternative to paper-based consent forms [42]. However, the possibility to provide electronic consent should not imply the abandonment of a thorough informative process.

### IC in research with vulnerable groups

As mentioned, the Declaration of Helsinki provides guidelines for IC in research with vulnerable groups, i.e. participants incapable of giving consent, minors, and emergency patients. Here differences between national regulations become visible. German and Polish regulations foresee the involvement of incapacitated participants or minors in the process of IC through their assent to additionally support the decision of guardians. Russian norms request only consent from parents, legal representatives, or guardians, thus hardly involving vulnerable research participants in the IC process. This could indicate a certain paternalistic approach, observable also in the research process in other post-communist countries of Central and Eastern Europe [7]. It also reveals a weak commitment to make use of communication techniques developed over the last decades that seek to enhance the participation of those with reduced or different cognitive abilities and minors. Specific to German regulations is the possibility of refusing participation in research through a simple verbal or bodily statement or expression [43].

### The need for national and international harmonization

The examination of norms pertaining to the question of IC shows that in each country this topic is repeatedly codified in several laws and regulations. A lack of coherent and transparent legislation can have a negative impact on the planning and implementation of clinical trials and creates legal uncertainty [44, 45]. Variations in legal regulations increase the time and resources required to obtain approvals for multinational and multicenter studies. As a result, sponsors and researchers in multinational and multicenter trials must have extensive knowledge of laws in force in a particular country to develop a valid study design and the procedures for informed consent. Moreover, differential treatment as a result of ambiguous rules may lead to mistrust and may provide loopholes that can be exploited by researchers and regulators at the cost of patient autonomy and research integrity.

Uniform binding IC regulations could expedite medical research. Such efforts for harmonization of national legislation have been attempted on the level of regional international organizations for example the European Union (EU) or the Eurasian Economic Union (EAEU). The introduction of the “EU Regulation No 536/2014 on clinical trials on medicinal products for human use” had the goal to harmonize and simplify procedures for clinical trials, especially in the case of multi-center international trials. [46]. Similarly, EAEU’s “Agreement on Uniform Principles and Rules for the Circulation of Medicines within the framework of the EAEU”, which applies to Russia, allow to conduct the preclinical and clinical studies with medicinal products in the Member States according to rules of good clinical practice. However, despite the introduction of uniform rules, national regulations still maintain their significance [47, 48]. It is noticeable that Regulation 536/2014 regards IC as an aspect of an intrinsically national nature, the regimentation of which should be left to the discretion of each member state. This indicates that no European consensus has been reached on the modalities of IC. A major challenge for the harmonization of regulations is that IC is oftentimes strongly anchored in the cultural background of the particular country [6]. In Poland and Russia, the traditional doctor-patient relationship plays an important role in the declaration of consent in the context of biomedical research. Patients generally trust their medical staff very strongly and, in case of informed consent, refrain from critical questions [49, 50]. Moreover, in both countries, families play an active role in managing individual health care [51]. This indicates that the ethical principle of informed consent is not always to be assessed as individual consent, but, in some traditional circles, also includes family members, even when the normative rules do not set any standards for such a situation. Here we need to recall that while the Declaration of Helsinki acknowledges that it may be appropriate to consult family members, it explicitly states that participation of those capable of consenting is conditional to their individual agreement (§25). The lack of international agreement on legal regulations strengthens the role of ethical guidelines, particularly the Declaration of Helsinki, as general codes of good scientific and professional conduct. Legal regulations cannot solve all ethical issues and oftentimes do not keep up with the newest developments in medical technology and treatment. Therefore, it is important to explore the role and potential of responsible Research Ethics Committees in approval of research protocols, preparation of ethical guidelines and encouraging good scientific practice.

## Conclusion

Although the normative framework for IC in all three countries generally adheres to the guiding principles of the Declaration of Helsinki, visible are differences between national regulations, especially regarding the method and content of information, the form of consent, and obtaining assent from subjects incapable of consent. The identified differences can negatively impact the conduct of international clinical studies and reveal the important directing role of international ethical guidelines. Due to cultural, political, and social differences, achieving global or even regional legislative harmonization might prove impossible—even within the closely interconnected Pan-European area. The lack of legal consensus reinforces the significance of the international ethical agreements. However, efforts for harmonization will remain unfulfilled if debates at the legal level are not complemented with an international discourse on ethical issues and an exchange of experiences among ethics committees.

There is a need for improvement in national legal regulations. Special attention needs to be paid, so that flexibilities within national regulations are not misused to undermine protection of participants, especially regarding the questions of IC. We can expect in biomedical research a similar risk as with other areas with high financial stakes. Countries may be tempted to offer progressively laxer regulations in a competition to attract foreign capital or to grant regulatory approval through dubious means at the cost of individual autonomy and research integrity. An erosion of standards should be confronted for both ethical and epistemic reasons. Failure to follow protocols may not only discourage transparency over IC practices, but also over research processes themselves. Furthermore, these divergent standards make it more difficult to assess whether research carried out in countries with different value systems showed the same level of concern for respecting the principle of IC. An important role here falls on national and local Research Ethics Committees, to oversee that the guidelines of the Declaration of Helsinki are adequately interpreted in context of national norms and cultural values and orientations. Future research needs to assess whether ethics committees have indeed the power to assist the legal apparatus in making sure the principle of IC is respected.

## Data Availability

All data analyzed during this study are included in this published article. Datasets used and analyzed in this study are publicly available.
